# Real-World Effectiveness and Optimal Dosage of Favipiravir for Treatment of COVID-19: Results from a Multicenter Observational Study in Thailand

**DOI:** 10.3390/antibiotics11060805

**Published:** 2022-06-15

**Authors:** Pinyo Rattanaumpawan, Supunnee Jirajariyavej, Kanokorn Lerdlamyong, Nattawan Palavutitotai, Jatuporn Saiyarin

**Affiliations:** 1 Division of Infectious Diseases and Tropical Medicine, Department of Medicine, Faculty of Medicine Siriraj Hospital, Mahidol University, Bangkok 10700, Thailand; 2 Internal Medicine Unit, Taksin Hospital, Bangkok 10600, Thailand; jsupunee@yahoo.com; 3 Internal Medicine Unit, Vachira Phuket Hospital, Phuket 83000, Thailand; ornmd@live.com; 4 Internal Medicine Unit, Lerdsin Hospital, Bangkok 10500, Thailand; nattawan.pala@gmail.com; 5 Internal Medicine Unit, Central Hospital, Bangkok 10100, Thailand; ooyaoi@hotmail.com

**Keywords:** COVID-19, favipiravir, pneumonia

## Abstract

Favipiravir is a broad-spectrum oral antiviral agent that shows in vitro activity against SARS-CoV-2. Presently, data on the real-world effectiveness and optimal dosage of favipiravir for treating COVID-19 are limited. We conducted a retrospective observational study of hospitalized adult patients with COVID-19 at five tertiary care hospitals in Thailand. We reviewed patient charts to obtain all necessary data. Among 247 COVID-19 patients, 63 (23.0%) received ≥1 dose of favipiravir. Of these 63 patients, 61.9% were male with a median age of 48 years (range 22–85 years), 27.0% required an O_2_ nasal cannula, 9.5% required non-invasive ventilation and/or high-flow O_2_ therapy, and 6.4% required invasive mechanical ventilation and/or ECMO. The median baseline NEWS2 score was 5 (0–16). The Day-7 clinical improvement rate [95%CI] was 66.7% [53.7–78.0%] in all patients, 92.5% [75.7–99.1%] in patients who did not require O_2_ supplementation, and 47.2% [0.4–64.5%] in patients who required O_2_ supplementation. No life-threatening adverse events were identified. The 28-day mortality rate was 4.8%. A multivariate analysis revealed three poor prognostic factors for Day-7 clinical improvement (odds ratio (95%CI); *p*-value): older age (0.94 (0.89–0.99); *p* = 0.04), a higher baseline NEWS2 score (0.64 (0.47–0.88); *p* = 0.006), and a lower favipiravir loading dose (≤45 mg/kg/day) (0.04 (0.005–0.4); *p* = 0.006). In conclusion, our study reports the promising effectiveness of favipiravir for treating COVID-19 patients. In addition to older age and a high baseline NEWS2 score, a low loading dose of favipiravir (≤45 mg/kg/day) was also identified as a poor prognostic factor for early clinical improvement. Further studies to explore the optimal dose and the optimal timing of drug initiation for favipiravir should be performed.

## 1. Introduction

As of 16 May 2022, over 519 million confirmed COVID-19 cases and over 6.2 million COVID-19-related deaths have been reported globally [1]. This pandemic disease is caused by a novel coronavirus named severe acute respiratory syndrome coronavirus 2 (SARS-CoV-2). SARS-CoV-2 is a single-stranded RNA beta-coronavirus encoding an RNA-dependent RNA polymerase (RdRp) and proteases. Both RdRp and viral proteases are considered important targets for potentially therapeutic agents. Thus far, remdesivir, molnupiravir, and nirmatrelvir/ritonavir have shown promising efficacy in landmark clinical trials [2,3,4,5,6]. Unfortunately, data on the efficacy and optimal dosage of favipiravir for treating COVID-19 are still limited.

Favipiravir, a purine nucleic acid analog, is a broad-spectrum oral antiviral agent that inhibits the RdRp of RNA viruses [7]. This agent shows in vitro activity against many RNA viruses, including arenaviruses, bunyaviruses, flaviviruses, Ebola virus, and influenza virus, as well as SARS-CoV-2 [7,8]. Several randomized control trials (RCTs) of favipiravir among COVID-19 patients have already reported their results [9,10,11,12,13,14,15,16,17,18,19]. One of the interesting studies was an open-label RCT of 150 patients with mild-to-moderate COVID-19, which revealed a significantly shorter median time to clinical cure in the favipiravir group compared with the standard supportive care group (3 days vs. 5 days; *p* = 0.03) [14]. Another interesting study was a RCT of 100 patients with symptomatic COVID-19 infections [8]. The study reported a comparable onset of SARS-CoV-2 PCR negativity in the favipiravir group compared with the hydroxychloroquine group (8.3 days vs. 8.1 days; *p* = 0.79) [13]. A recent meta-analysis of 17 clinical trials of favipiravir reported the significant benefit of favipiravir on clinical and virological outcomes among hospitalized patients [20]. Additionally, there was no statistically significant difference in the mortality rate between the favipiravir group and the control group [20]. Favipiravir seems to be more beneficial among patients with mild-to-moderate COVID-19 and patients who received early favipiravir therapy [20]. The benefits of early favipiravir initiation in mild COVID-19 cases are in need of further investigation.

In February 2020, favipiravir was made available for use in Thailand under emergency procurement by the Department of Disease Control of Thailand. In early March 2020, the Thailand National Clinical Practice Guidelines (CPG) for COVID-19 management recommended the initiation of favipiravir therapy only in patients with severe COVID-19 pneumonia (pneumonia with required high-flow O_2_ supplementation, non-invasive mechanical ventilation, or invasive mechanical ventilation to maintain a patient O_2_ saturation of 90% or more). In May 2020, the national CPGs were revised, and favipiravir was also recommended for the treatment of mild pneumonia (i.e., abnormal chest X-ray without desaturation). As of May 2022, the national CPGs no longer recommend favipiravir for the treatment of pneumonia. The first-line therapy for COVID-19 pneumonia is remdesivir. Favipiravir therapy is now only recommended for the treatment of mild COVID-19 infections.

The standard dose of favipiravir for treating an influenza infection is 1600 mg twice daily on Day 1, followed by 600 mg twice daily on Days 2 to 5 [21]. A maximal loading dose of 3000 mg twice daily on Day 1 and a maintenance dose of 1200 mg twice daily on Days 2–9 were safely used in a previous Ebola study [22]. In the previous COVID-19 clinical trials, the loading dose varied from 1600 to 1800 mg twice daily on Day 1, and the maintenance dose ranged from 600 to 800 mg twice daily afterward [9,10,11,12,13,14,15,16,17,18,19]. Given that the optimal dose of favipiravir for treating COVID-19 is still uncertain, the 2020 Thailand National Clinical Practice Guidelines (CPG) recommended a fixed loading dose of 1600 mg twice daily on Day 1, followed by 600 mg twice daily on Days 2 to 10. A higher loading dose (60 mg/kg/day, MKD) and maintenance dose (20 MKD) are recommended in patients with a body mass index (BMI) of ≥35.

Presently, data on the effectiveness and optimal dosage of favipiravir for treating COVID-19 are limited. Therefore, we conducted a study to explore these issues.

## 2. Materials and Methods

### 2.1. Study Design

We conducted a retrospective observational study of COVID-19 patients who were hospitalized at any of the five tertiary care hospitals in Thailand (Siriraj, Taksin, Vachira Phuket, Lerdsin, and Central hospitals) during the period of 1 January to 30 April 2020. The study protocol was approved with a waiver of informed consent by the institutional review boards of all involved hospitals.

### 2.2. Inclusion and Exclusion Criteria

We enrolled all hospitalized patients of at least 18 years of age who had reverse transcription PCR-confirmed SARS-CoV-2 based on a respiratory specimen (nasopharyngeal, oropharyngeal, sputum, endotracheal aspirate, or bronchoalveolar lavage sample) and received at least one dose of favipiravir. Patients who expired or were discharged from the hospital within 24 h after hospitalization were excluded.

### 2.3. Data Collection and Study Definition

We reviewed patient charts to obtain all necessary data, including demographic data, clinical data, laboratory data, and the hospital stay length. We also recorded the daily National Early Warning Score 2 (NEWS2 score). Details regarding the NEWS2 score have been published elsewhere [11]. The primary outcome was the rate of clinical improvement within seven days of favipiravir therapy (Day-7 clinical improvement), and the secondary outcomes were the Day-14 and Day-28 clinical improvement rates.

Clinical improvement was defined as a one-point reduction in baseline status (on the first day of favipiravir therapy) on a six-point disease severity scale at the time of evaluation. The six-point disease severity scale was categorized as follows: 6—death; 5—hospitalization for extracorporeal membrane oxygenation (ECMO) or mechanical ventilation; 4—hospitalization for non-invasive ventilation or high-flow O_2_ therapy; 3—hospitalization for supplemental O_2_; 2—hospitalization without the need for O_2_ supplementation but requiring ongoing medical care; and 1—discharge or normalization of all vital signs and saturation of peripheral O_2_ of >94% on room air for at least 24 h.

### 2.4. Statistical Analysis

Categorical variables are summarized by frequency and percentage, while continuous variables are summarized by the median and range. Univariate analyses were performed using the Fisher exact test for categorical data. The Mann–Whitney U test was used for continuous data. To identify the factors independently associated with the Day-7 clinical improvement, we performed a subsequent multivariate analysis including all potentially significant variables with a *p*-value of ≤0.20 in a stepwise fashion.

For all calculations, a two-tailed *p*-value of <0.05 was considered statistically significant. All calculations were performed using STATA version 14.1 (Stata Corp, College Station, TX, USA).

## 3. Results

During the study period, there were a total of 274 COVID-19 patients hospitalized in the participating hospitals, of which 63 patients (23.0%) received favipiravir. The baseline demographics and characteristics of all patients are listed in Table 1.

The median age of favipiravir-treated COVID-19 patients was 48 (22–85) years, and 39 of these patients (61.9%) were male. Most patients had a fever (87.3%), a sore throat (69.8%), or a cough (74.6%) as the clinical presentation. The median duration between the symptom onset and the admission date was 6 (0–28) days, while the median duration between the symptom onset and the first day of favipiravir therapy was 8 (0–28) days.

At baseline (Day 1 of favipiravir therapy), 17 patients (27.0%) required O_2_ supplementation via nasal cannula, 6 patients (9.5%) required non-invasive ventilation and/or high-flow O_2_ therapy, and 4 patients (6.4%) required invasive mechanical ventilation and/or ECMO, while the remainder did not require O_2_ supplementation. The median baseline NEWS2 score was 5 (0–16).

The median loading dose of favipiravir was 47.4 (29.1–71.1) mg/kg/day, and one-third of enrolled patients (33.3%) received a loading dose of ≤45 mg/kg/day. The median maintenance dose of favipiravir was 17.9 (10.9–26.7) mg/kg/day, and 76.2% of the subjects received a maintenance dose of ≤15 mg/kg/day. The median duration of favipiravir therapy was 12 (2–17) days. Within two days of initiating favipiravir treatment, nearly all patients were prescribed a chloroquine-based agent (98.4%) and a protease inhibitor (96.8%); half of them also received azithromycin (49.2%). Only few received a steroid (12.7%) or tocilizumab (6.4%).

### 3.1. Hospital Course and Treatment Outcomes

**Figure 1:**
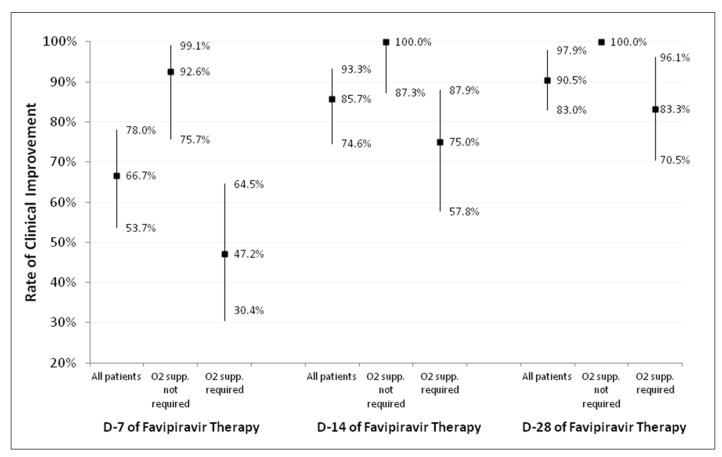
Rate of clinical improvement on Day 7, Day 14, and Day 28 of favipiravir therapy, stratified by the requirement for O_2_ supplementation.

Details regarding the hospital course and treatment outcomes are shown in Table 2. The Day-7, Day-14, and Day-28 clinical improvement rates, stratified by the requirement for O_2_ supplementation, are depicted in Figure 1. The Day-7 clinical improvement rate [95%CI] was 66.7% [53.7–78.0%] in all patients, 92.5% [75.7–99.1%] in patients who did not require O_2_ supplementation (a six-point disease severity scale score of 1–2), and 47.2% [0.4–64.5%] in patients who required O_2_ supplementation (a six-point severity scale score of 3–5). The Day-14 clinical improvement rates for all patients, those who did not require O_2_ supplementation, and those who required O_2_ supplementation were 85.7% [74.6–93.2%], 100.0% [87.2–1.00%], and 75.0% [57.8–87.9%], respectively. Nearly all patients who required O_2_ supplementation (96.1%) had clinical improvement within 28 days.

Of the 63 favipiravir-treated patients, 4 patients required invasive mechanical ventilation or ECMO on Day 1 of therapy, and 4 more cases subsequently required invasive mechanical ventilation (two cases on Day 6 and two cases on Day 9 of therapy). The 14-day, 28-day, and in-hospital mortality rates were 1.6%, 4.8%, and 7.9%, respectively. The major cause of death was superimposed infection.

The most common adverse event was diarrhea (54.0%), followed by nausea/vomiting (7.9%), hepatitis (6.4%), and QT interval prolongation in electrocardiogram (6.4%). None of these adverse events were life-threatening.

### 3.2. Factors Associated with Day-7 Clinical Improvement

To determine the factors associated with Day-7 clinical improvement, we compared the patients with Day-7 clinical improvement (cases) with the patients without Day-7 clinical improvement (controls). The characteristics of both groups are shown in Table 1. The cases had a significantly lower age (47 vs. 59 years; *p* = 0.02), a significantly lower BMI (25.0 vs. 27.9; *p* = 0.04), a significantly lower baseline NEWS2 score (4 vs. 5; *p* = 0.003), and a significantly lower baseline six-point disease severity scale score (2 vs. 3; *p* < 0.001). Additionally, the baseline white blood cell count was significantly lower in the case group (5420 vs. 6810; *p* = 0.03). Although the median loading and maintenance doses of favipiravir were not statistically different between these groups, the proportion of patients in the control group who received a lower loading dose of favipiravir (≤45 MKD) trended higher compared to the case group (26.2% vs. 47.6%; *p* < 0.10).

Table 3 shows the results of the multivariate analysis. A multivariate analysis revealed three factors that were negatively associated with Day-7 clinical improvement (odds ratio (95%CI); *p*-value): older age (0.94 (0.89–0.99); *p* = 0.04), higher baseline NEWS2 score (0.64 (0.47–0.88); *p* = 0.006), and a lower loading dose of favipiravir (≤45 MKD) (0.04 (0.005–0.4); *p* = 0.006).

## 4. Discussion

Our study enrolled a total of 63 COVID-19 patients who received ≥1 dose of favipiravir. Of these 63 patients, most of them had moderate-to-severe pneumonia with a median baseline NEWS2 score of 5. The Day-7 clinical improvement rate [95%CI] was 66.7% [53.7–78.0%] in all patients, 92.5% [75.7–99.1%] in patients who did not require O_2_ supplementation, and 47.2% [0.4–64.5%] in patients who required O_2_ supplementation. The most common adverse event was diarrhea (54.0%). No life-threatening adverse events were identified. The 28-day mortality rate was 4.8%.

The Day-7 clinical improvement rate from our study was 67.7%, which is slightly lower than the Day-7 clinical recovery rate from the abovementioned RCT of favipiravir (71.4%) [13]. However, there were a few differences between these two studies. First, the definition of clinical recovery used in the abovementioned RCT was based mainly on clinical symptoms (e.g., fever, cough), whereas the definition of clinical improvement used in our study was based on improvement in oxygenation status. Our study included sicker patients with a higher proportion of patients who required mechanical ventilation (6.4%) as compared with the subjects of the unpublished RCT (0.9%). These differences may explain the slightly lower rate of favorable clinical responses observed in the present study.

Among the COVID-19 patients who did not require O_2_ supplementation, nearly all patients (92.6%) had clinical improvement within the first seven days of favipiravir therapy. However, only half of the patients who required O_2_ supplementation (47.2%) had clinical improvement within the first seven days of therapy. The rate of clinical improvement in these patients finally reached 75% on Day 14 and 83.3% on Day 28. Of the eight patients who required invasive mechanical ventilation or ECMO during their hospitalization, one patient died within the first 14 days. Therefore, the calculated 14-day mortality among this group was 12.5%. This number is similar to the 14-day mortality reported by a remdesivir RCT, in which 13 (10.4%) out of 125 patients who required mechanical ventilation or ECMO died [2]. Based on these findings, the effectiveness of favipiravir for treating COVID-19 is promising, but this drug may be ineffective in more severe cases.

Our study identified older age and a higher baseline NEWS2 scale as poor prognostic factors for early clinical response. These findings are compatible with the results from many previous publications [23,24,25]. We also explored other baseline variables (e.g., BMI, comorbidities); however, the impact of those factors disappeared after the data were adjusted by the baseline NEWS2 scale.

Given that the optimal dose of favipiravir is still uncertain, we carefully explored the association between favipiravir dosage and patient outcome. Our study confirmed that a loading dose of favipiravir of ≤45 mg/kg/day was a poor prognostic factor for early clinical response. Therefore, a fixed favipiravir loading dose of 1600 mg twice daily for all patients with a BMI of <35 may be suboptimal for patients with a BMI of <35 but a body weight of ≥70 kg. Some might argue that this significant association may be a reflection of patients’ obesity, which was also known as a poor prognostic factor in COVID-19. However, our study did not find any association between the patients’ baseline BMI or body weight and the treatment outcome in the multivariate analysis. Based on these findings, the recommended dosages of favipiravir in Thailand were revised in January 2021. Among those with a body weight of less than 80 kg, the recommended dose is 1800 mg twice daily on Day 1 and 800 mg twice daily on Days 2 to 10. Among those with a body weight of 80 kg or more, the recommended dose is 2400 mg twice daily on Day 1 and 1000 mg twice daily on Days 2 to 10.

Our study has several strengths. First, this study was a very early study to explore the real-world effectiveness of favipiravir in active clinical cases of COVID-19. Second, this study included patients with differing disease severities; the patients ranged from mild pneumonia cases who did not require O_2_ supplementation to patients with life-threatening pneumonia who required mechanical ventilation or ECMO. This diverse subject pool provided us with some information regarding the effectiveness of favipiravir and the clinical course of COVID-19 disease in various degrees of severity. Lastly, the daily NEWS2 scores and six-point disease severity scale scores were carefully collected and analyzed. Consequently, we can report nearly all important clinical outcomes and compare our findings with those of other clinical trials [2,13,14,17].

Our study also has some limitations. First, the retrospective design resulted in a significant amount of missing data, especially for laboratory values. To resolve this issue, when performing the multivariate analysis, missing data were replaced by the mean value of a given variable. Second, the majority of our patients also received a chloroquine-based agent and protease inhibitors. Therefore, the adverse drug reactions among our patients may be affected by these medications. A high rate (54.0%) of diarrhea in this study was probably related to a protease inhibitor rather than favipiravir. Third, a sample size of 63 patients with COVID-19 pneumonia is not large enough to detect other associated factors with a low prevalence. Lastly, the generalizability of our findings may be an issue. Given that the study was conducted in tertiary care hospitals in Thailand, results may not be applicable to COVID-19 patients in different settings.

In conclusion, our descriptive study reports the favorable effectiveness of favipiravir for treating COVID-19 patients in a tertiary care hospital setting. No life-threatening adverse events were identified. In addition to older age and a high baseline NEWS2 score, a low loading dose of favipiravir (≤45 mg/kg/day) was also identified as a poor prognostic factor for early clinical improvement. Based on these findings, a higher loading dose of favipiravir may be necessary. Further studies to explore the optimal dose and the optimal timing of drug initiation for favipiravir should be performed.

## Figures and Tables

**Table 1:** Baseline demographics and characteristics of all patients.

Variables	All (n = 63)	Day-7 Clinical Improvement		*p*-Value
		Yes (n = 42)	No (n = 21)	
Age, median (range), year	48 (22–85)	47 (23–72)	59 (22–85)	0.02
Male sex	39 (61.9%)	25 (59.5%)	14 (66.7%)	0.78
Body weight, median (range), kg	69 (45–125)	68 (51–125)	76 (45–120)	0.08
Body mass index median (range), kg/m^2^	26.1 (19.0–43.8)	25.0 (19.0–43.8)	27.9 (20.8–39.2)	0.04
**Duration between, median (range), day**				
Symptom onset and admission date	6 (0–28)	6 (0–28)	8 (0–15)	0.08
Admission date and Day 1 of favipiravir therapy	1 (−8–10)	1 (−3–10)	0 (-8–5)	0.002
Symptom onset and Day 1 of favipiravir therapy	8 (0–28)	8 (2–28)	8 (0–11)	0.60
**Exposure risk**				
Contact with confirmed COVID-19 cases	26 (41.3%)	19 (45.2%)	7 (33.3%)	0.42
Travel abroad	7 (11.1%)	5 (11.2%)	2 (9.5%)	1.00
Contact with a foreigner	11 (17.5%)	8 (19.1%)	3 (14.3%)	0.74
Travel to a local area with clustered cases	38 (60.3%)	28 (66.7%)	10 (47.6%)	0.18
**Underlying diseases**				
Heart disease and hypertension	9 (14.3%)	7 (16.7%)	2 (9.5%)	0.71
Diabetes mellitus	17 (27.0%)	11 (26.2%)	6 (28.6%)	1.00
Chronic lung disease	4 (6.4%)	2 (7.1%)	1 (4.8%)	1.00
Chronic kidney disease	4 (6.4%)	3 (7.1%)	1 (4.8%)	1.00
Chronic liver disease	3 (4.8%)	3 (7.1%)	0 (0%)	0.55
Solid cancer	4 (6.4%)	2 (7.1%)	1 (4.8%)	1.00
Others	4 (6.4%)	2 (7.1%)	2 (9.5%)	0.60
**Clinical presentation upon admission**				
Fever or body temperature of >37.5 °C	55 (87.3%)	36 (85.7%)	19 (90.5%)	0.71
Sore throat	44 (69.8%)	27 (64.3%)	17 (81.0%)	0.25
Rhinorrhea	16 (25.4%)	13 (31.0%)	3 (14.3%)	0.22
Cough	47 (74.6%)	30 (71.4%)	17 (81.0%)	0.54
Headache	11 (17.5%)	8 (19.1%)	3 (14.3%)	0.74
Myalgia	17 (27.0%)	12 (28.6%)	5 (23.8%)	0.77
Diarrhea	8 (12.7%)	6 (14.3%)	2 (9.5%)	0.71
Shortness of breath	27 (42.9%)	14 (33.3%)	13 (61.9%)	0.06
**Illness severity at the time of favipiravir initiation**				
NEWS2 score, median (range)	5 (0–16)	4 (0–11)	5 (0–16)	0.003
Six-point disease severity scale, median (range)	2.5 (1–5)	2 (1–4)	3 (2–5)	<0.001
1—No O_2_ supplementation with O_2_ saturation >94%	4 (6.4%)	4 (6.4%)	0 (0)	<0.001
2—No O_2_ supplementation with O_2_ saturation ≤94%	23 (36.4%)	21 (50.0%)	2 (9.5%)	
3—Requiring O_2_ supplementation	28 (44.4%)	16 (40.1%)	12 (57.1%)	
4—Requiring high-flow O_2_ supplementation or non-invasive mechanical ventilation	4 (6.4%)	1 (2.4%)	3 (14.3%)	
5—Requiring invasive mechanical ventilation and/or extracorporeal membrane oxygenation	4 (6.4%)	0 (0%)	4 (19.1%)	
Baseline laboratory values *				
Hemoglobin, median (range), (mg/dl)	14.0 (8.0–18.0)	14.0 (9.0–17.0)	13.5 (8.0–18.0)	0.48
White blood cell count, median (range), (cell/mm^3^)	5735 (2910–41300)	5420 (2910–41300)	6810 (3180–15750)	0.03
Serum creatinine, median (range), (mg/dl)	0.9 (0.3–22.9) (n = 58)	0.9 (0.4–22.9) (n = 27)	0.9 (0.33–5.1) (n = 21)	0.67
Serum albumin, median (range), (mg/dl)	4.0 (1.8–4.9) (n = 53)	4.2 (1.8–5.0) (n = 33)	3.5 (2.6–4.1) (n = 20)	0.002
Serum lactate dehydrogenase, median (range), (mg/dl)	404 (145–1094) (n = 30)	382 (145–567) (n = 17)	453 (313–1094) (n = 13)	0.03
**Indication of favipiravir therapy**				
Abnormal chest imaging only	26 (41.3%)	24 (57.1%)	2 (9.5%)	<0.001
Required O_2_ supplementation only	3 (4.7%)	2 (4.7%)	1 (4.8%)	
Abnormal chest X-ray and required O_2_ supplementation	34 (54.0%)	16 (38.1%)	18 (85.7%)	
Favipiravir regimen				
Dose per body weight, median (range), mg/kg/day				
Loading dose	47.4 (29.1–71.1)	49.2 (29.1–62.7)	45.7 (29.6–71.1)	0.47
Maintenance dose	17.9 (10.9–26.7)	18.5 (10.9–23.5)	17.1 (11.1–26.7)	0.37
Potentially sub-therapeutic dose				
Loading dose of ≤45 MKD	21 (33.3%)	11 (26.2%)	10 (47.6%)	0.10
Maintenance dose of ≤15 MKD	48 (76.2%)	33 (78.6%)	15 (71.4%)	0.55
Duration of therapy, median (range), day	12 (2–17)	11.5 (2–16)	12 (2–17)	0.02
Other medications used **				
Any chloroquine-based agent	62 (98.4%)	41 (97.6%)	21 (100%)	1.00
Hydroxychloroquine	54 (85.7%)	36 (85.7%)	18 (85.7%)	1.00
Chloroquine	14 (22.2%)	8 (19.1%)	6 (28.6%)	0.52
Any protease inhibitor	61 (96.8%)	40 (95.2%)	21 (100,0%)	0.55
Darunavir/ritonavir	51 (81.0%)	35 (83.3%)	16 (76.2%)	0.51
Lopinavir/ritonavir	22 (34.9%)	13 (31.0%)	9 (42.9%)	0.26
Azithromycin	31 (49.2%)	17 (40.5%)	14 (66.7%)	0.06
Steroid	8 (12.7%)	5 (11.9%)	3 (14.3%)	1.00
Tocilizumab	4 (6.4%)	1 (2.4%)	3 (14.3%)	0.10

Note. * Earliest results of a test obtained within the first 7 days of admission (missing data were replaced by the mean value of the variable). ** Medications used within 2 days before or after the initiation of favipiravir therapy.

**Table 2:** Hospital course and treatment outcomes.

Variables	All Patients (n = 63)
Clinical improvement	
Day-7 clinical improvement	42 (66.7%)
Patients who did not require O_2_ supplementation (n = 27)	25 (92.6%)
Patients who required O_2_ supplementation (n = 36)	17 (47.2%)
Day-14 clinical improvement	54 (85.7%)
Patients who did not require O_2_ supplementation (n = 27)	27 (100.0%)
Patients who required O_2_ supplementation (n = 36)	27 (75.0%)
Day-28 clinical improvement	57 (90.5%)
Patients who did not require O_2_ supplementation (n = 27)	27 (100.0%)
Patients who required O_2_ supplementation (n = 36)	30 (83.3%)
ICU duration, median (range), day	0 (0–46)
Required IMV * or ECMO ** during hospitalization	8 (12.7%)
Required IMV * or ECMO ** before initiation of favipiravir	4 (6.3%)
Required IMV * or ECMO ** after initiation of favipiravir	4 (6.3%)
14-day mortality rate	1 (1.6%)
28-day mortality rate	3 (4.8%)
In-hospital mortality rate	5 (7.9%)
Length of hospital stay, median (range), day	15 (2–47)
**Adverse drug reactions**	39 (61.9%)
Diarrhea	34 (54.0%)
Hepatitis	4 (6.4%)
QT interval prolongation	4 (6.4%)
Nausea and vomiting	5 (7.9%)
Superimposed bacterial infection	8 (12.7%)

Note. * IMV: invasive mechanical ventilation; ** ECMO: extracorporeal membrane oxygenation.

**Table 3:** Factors associated with Day-7 clinical improvement.

Variables	Unadjusted Odd Ratio [95%CI; *p*-Value]	Adjusted Odd Ratio [95%CI; *p*-Value]
Age, year	0.95 [0.92–099; *p* = 0.02]	0.94 [0.89–0.99; *p* = 0.04]
Baseline NEWS2 score	0.77 [ 0.65–0.92; *p* = 0.004]	0.64 [0.47–0.88; *p* = 0.006]
Low loading dose of favipiravir	0.39 [0.13–1.17; *p* = 0.09]	0.04 [0.005–0.41; *p* = 0.006]

## Data Availability

The dataset generated and/or analyzed during the current study are available from the corresponding author on reasonable request.
